# Selective inhibition of carbonic anhydrase-IX by sulphonamide derivatives induces pH and reactive oxygen species-mediated apoptosis in cervical cancer HeLa cells 

**DOI:** 10.1080/14756366.2018.1481403

**Published:** 2018-07-12

**Authors:** Ismail Koyuncu, Ataman Gonel, Abdurrahim Kocyigit, Ebru Temiz, Mustafa Durgun, Claudiu T. Supuran

**Affiliations:** a Department of Biochemistry, Faculty of Medicine, Harran University, Sanliurfa, Turkey;; b Department of Medical Biochemistry, Faculty of Medicine, Bezmialem Vakif University, Istanbul, Turkey;; c Department of Chemistry, Faculty of Arts and Sciences, Harran University, Sanliurfa, Turkey;; d Laboratorio di Chimica Bioinorganica, Università degli Studi di Firenze, Polo Scientifico, Sesto Fiorentino, Florence, Italy;; e Neurofarba Department, Section of Pharmaceutical and Nutriceutical Sciences, Università degli Studi di Firenze, Sesto Fiorentino, Florence, Italy

**Keywords:** Carbonic anhydrase IX, sulphonamide, apoptosis, reactive oxygen species, anticancer agent

## Abstract

Selective inhibition with sulphonamides of carbonic anhydrase (CA) IX reduces cell proliferation and induces apoptosis in human cancer cells. The effect on CA IX expression of seven previously synthesised sulphonamide inhibitors, with high affinity for CA IX, as well as their effect on the proliferation/apoptosis of cancer/normal cell lines was investigated. Two normal and three human cancer cell lines were used. Treatment resulted in dose- and time-dependent inhibition of the growth of various cancer cell lines. One compound showed remarkably high toxicity towards CA IX-positive HeLa cells. The mechanisms of apoptosis induction were determined with Annexin-V and AO/EB staining, cleaved caspases (caspase-3, caspase-8, caspase-9) and cleaved PARP activation, reactive oxygen species production (ROS), mitochondrial membrane potential (MMP), intracellular pH (pHi), extracellular pH (pHe), lactate level and cell cycle analysis. The autophagy induction mechanisms were also investigated. The modulation of apoptotic and autophagic genes (Bax, Bcl-2, caspase-3, caspase-8, caspase-9, caspase-12, Beclin and LC3) was measured using real time PCR. The positive staining using γ-H2AX and AO/EB dye, showed increased cleaved caspase-3, caspase-8, caspase-9, increased ROS production, MMP and enhanced mRNA expression of apoptotic genes, suggesting that anticancer effects are also exerted through its apoptosis-inducing properties. Our results show that such sulphonamides might have the potential as new leads for detailed investigations against CA IX-positive cervical cancers.

## 1. Introduction

The majority of current research on cancer treatments is targeted on the development of specific methods and treatment techniques. Promising results have emerged from recent studies on cancer treatment using carbonic anhydrase IX (CA IX) enzyme inhibitors^1–3^. CA IX causes tumour cell growth by preventing acidosis associated with hypoxia through the alkalinisation of intracellular pH (pHi) and an increase in metastatic activity by creating an extracellular acidosis (pHe) environment^4^^,^^5^. These findings show that CA IX could be a useful therapeutic target against cancer^6^. Therefore, there is significant evidence that an intervention to be made to one or more effectors of this enzyme could have an inhibiting effect on tumour growth^7^^,^^8^.

CA IX enzyme is a very well-known transmembrane enzyme that is synthesised at a high rate in several solid cancer types and in more limited amounts in several normal tissues. Moreover, in several clinical studies, a clear relationship has been shown between high levels of CA IX and a poor prognosis^3^^,^^9^. This feature therefore makes CA IX a clinically suitable new target for cancer treatment^1–3^^,^^10^.

In recently studies, various sulphonamide derivative inhibitors of CA IX have been shown to halt cancer cell proliferation in an *in vitro* environment and to be effective in the reduction of tumour growth and have been determined to inhibit metastasis without any nonspecific toxic effects in various tumour models^3^^,^^11^. In addition, when these types of inhibitors have been applied, especially in conventional chemotherapy or in combination with radiotherapy, they have been shown to inhibit the growth of various tumours^7^^,^^11–15^.

In a previous study, we have demonstrated the synthesis and inhibitory activity against carbonic anhydrase isoforms I, II, IX and XII of some sulphonamide derivatives. In this study, the cytotoxic effects were examined on cancer cells and normal cells of CA IX expression of seven synthesised sulphonamide derivatives determined with the CA IX inhibitor property. In addition, by examining the effects on cell proliferation, apoptosis and autophagy of compounds showing a high cytotoxic effect, it was aimed to investigate the underlying molecular mechanisms of the potential antitumour effect of CA IX inhibitors.

## 2. Materials and methods

The cell culture medium (RPMI 1640), DMEM-F12, foetal bovine serum (FBS), streptomycin and penicillin were purchased from Gibco BRL (Life Technologies, Paisley, Scotland); WST-1 (Roche, Germany), ROS kit (Abcam, Cambridge, UK), MPP kit, ethidium bromide, acridine orange, trypsin–EDTA solution and dimethyl sulphoxide (DMSO), from Sigma Chemical Company (Germany) and the culture plates from Nunc (Brand Products, Denmark).

### 2.1. Cell culture and drugs

Cancer and normal cell lines were purchased from ATCC and stored in liquid nitrogen. HT-29 (colon adenoma cancer), HeLa (cervix adenoma cancer cell), MDA-MB-231 (breast adenoma cancer cell), HEK-293 (embryonic kidney epithelial cell) and PNT-1A (normal prostate cells) cell lines were incubated in DMEM: F-12 and RPMI-1640, including 10% foetal bovine serum (FBS), 100 µg/mL streptomycin/100 IU/mL penicillin, at 37 °C in an incubator containing 5% CO_2_, 95% air in a humid atmosphere.

The CA inhibitor aromatic sulphonamides used in this research were obtained according to our previous study. Briefly, the sulphonamide derivatives were synthesised through the reaction of 4-aminobenzenesulphonamide or 4-(2-aminoethyl) benzenesulphonamide with substituted aromatic aldehydes with catalytic amounts of formic acid in methanol at the refluxing temperature for 3–5 h. All the synthesised compounds were characterised with both analytical and spectral data. The aromatic aldehydes used in the synthesis were 5-bromo-2-hydroxybenzaldehyde^1^, 2-hydroxy-3-methylbenzaldehyde^2^^,^^3^, 4-methylbenzaldehyde^4^^,^^5^ and 4-methoxybenzaldehyde^6^^,^^7^. These CA inhibitors have been shown to induce a moderately effective, reversible inhibition of the membrane-bound isozyme CA IX compared with traditional inhibitors. The *K*i’s of the CA inhibitors and the chemical structures of the inhibitors tested are shown in Table 1^16^^,^^17^.

**Table 1.** Structures and K_i_ values of compounds^16^^,^^17^.


						*K_i_* (nM)			
Compound	n	–R_1_	–R_2_	–R_3_	–R_4_	hCA I	hCA II	hCA IX	hCA XII
**1**	2	–OH	–H	–H	–Br	351	413	187	397
**2**	2	–OH	–CH_3_	–H	–H	448	619	295	1107
**3**	0	–OH	–CH_3_	–H	–H	463	264	465	11.6
**4**	2	–H	–H	–CH_3_	–H	308	433	123	1084
**5**	0	–H	–H	–CH_3_	–H	>50.000	469	23.7	9.9
**6**	0	–H	–H	–OCH_3_	–H	>50.000	464	883	11.2
**7**	2	–H	–H	–OCH_3_	–H	442	600	231	1045

### 2.2. Cytotoxicity analysis

The cytotoxic effects of the substances were evaluated with WST-1 kits (Roche, Germany) in accordance with the manufacturer’s protocols. The cells were planted in 96-well plates (10^4^ cells in each well). After incubation for 24 h, the media were discarded and the substances and 5-Fu as the control drug, at doses of 0, 2.5, 5, 10, 25, 50, 100 and 200 µM, were incubated for 24, 48 and 72 h. WST-1 reactive of 10 µL was added to all wells. Following 4-h incubation, the measurements were taken on a plate reader (Spectramax M5) at wavelengths of 450 and 630 nm. Graphs were then created and the IC_50_ value of each substance was calculated.

### 2.3. Investigation of antiproliferative effects

The effects of the substances on the proliferation of HeLa cells were investigated using a commercial proliferation kit BrdU (BioVision, Germany) according to the manufacturer’s protocols. After 24 h, the cells were planted in 96-well plates (10^4^ cell/mL), the medium was replaced and the substances were administered at doses ranging from 2.5 to 200 µM. After 72 h, the media were discarded, 100 µL 1X of BrdU reactive was added and the samples were incubated in an incubator, containing 5% CO_2_, 95% fresh air, at 37 °C for 4 h. The medium was then removed, and 100 µL of fixative/denaturation solution was added and incubated for 30 min. 100 µL 1X of the antibody solution BrdU was added and, following 1-h incubation, was washed twice with 300 µL 1X of the washing solution. 100 µL 1X of anti-mouse HRP-linked antibody was added and incubated at room temperature for 1 h. Then, the cells were washed three times with 300 µL 1X of washing solution. The final incubation was performed with 100 µL of TMB substrate solution for 5 min, after which the stop solution was added, and then the reading was taken at a wavelength of 450 nm.

### 2.4. Apoptosis detection by Annexin-V FITC

The apoptotic effect of the substances was explored with the commercial FITC Annexin-V Apoptosis Detection Kit I (BD Biosciences, New Jersey, USA) according to the manufacturer’s protocol. The cells were planted in 6-well plates (5 × 10^5^ cells/well), and following 24-h incubation, sulphonamide derivative compounds at concentrations of 10–25–50 µM were administered, followed by incubation for 24 h. The cells were raised by trypsin handling and transferred into new tubes within 1X binding buffer, as 1 × 10^6^ in each tube, which were incubated for 15 min at room temperature. 5 µL of fluorochrome-conjugated Annexin-V and 5 µL of propidium iodide dyes were then added. 100 µL 1X binding buffer was added to the cells, and they were then centrifuged at 1200 rpm, for 5 min. Finally, the cells were analysed in flow cytometry (BD Via, New Jersey, USA).

### 2.5. Detection of intracellular pH (pHi), extracellular pH (pHe) and lactate level

Intracellular PH was measured according to the protocol of the fluorometric intracellular pH assay kit (Sigma, Germany, MAK-150). Fluorescent BCFL-AM indicator that was able to pass through the cell membrane was used to measure pHi fluctuations in the fluorometric intracellular pH kit. BCFL-AM was utilised to measure the reductions in pHi of the cells that had been treated by various conditions. The cells were planted in black plates, as 6 × 10^4^ in each well of the plate, and incubated for 24 h. The medium was replaced with BCFL-AM reagent which had been prepared in 100 µL of HBS solution (Hank’s buffer with 20 mM HEPES, 5 mM probenecid), then the cells were incubated at 37 °C in an atmosphere of 5% CO_2_ for 30 min (protected from light). Sulphonamide compound at doses of 0, 10, 25 and 50 µM was added to the HBS solution. In the next 5 min, the measurement was performed at wavelengths of 490 nm (excitation) and 535 nm (emission) in spectrofluorimetry (Spectramax, M5). Extracellular pH (pHe) and lactate levels were measured with commercial kit by the blood gas device (ABL90 FLEX PLUS, Radiometer, Copenhagen, Denmark) which is routinely used in the clinical biochemistry laboratory of hospital. After the application of the substance in this method, the media were analysed directly by the device.

### 2.6. Detection of intracellular ROS production

Intracellular free radicals were measured using the Cellular Reactive Oxygen Species Detection Assay Commercial Kit (Red Fluorescence, Abcam, Cambridge, UK, 186027). In this kit, a fluorescence probe that is permeable to the cell is used. When the probe reacts with ROS, it produces red fluorescence. The process was applied following kit protocol. The cells were planted in black plates, as 3 × 10^4^ in 100 µL, and incubated for 24 h. Sulphonamide compounds in PBS were administered at doses of 0–200 µM and incubated at 37 °C in an atmosphere of 5% CO_2_ for 1 h (protected from light). After the compounds were poured out, the wells were filled with 100 µL of ROS Deep Red Working Solution and incubated at 37 °C in an atmosphere of 5% CO_2_ for 1 h. A fluorometry device (Spectramax, M5) Ex/Em = 650/675 nm (cut off = 665 nm) was used to take the measurements.

### 2.7. Detection of mitochondrial membrane potential (MMP)

Mitochondrial membrane potential (MMP) changes appearing in the intrinsic pathway of mitochondria were spectrophotometrically detected. MMP was measured in a fluorometry device (Spectramax, M5), at wavelengths of 490 nm (excitation) and 530 nm(emission) according to the fluorometric Mitochondria Staining Kit (Sigma, Germany, CS0390) protocol.

### 2.8. Cell cycle assay

Cell cycle analysis was performed using BD Cycletest^™^ Plus DNA Reagent kit (BD Biosciences). According to the kit protocol, 1 × 10^6^ cells were planted in 6-well plates, and following 24 h, the cells were incubated for 24 h, with sulphonamide derivative compounds at concentrations of 10–25–50 µM. The cells were raised with trypsin handling and centrifuged at 1500 rpm for 5 min. A suspension was formed with 1X binding buffer, and respectively, 250 µL solution A was added and incubated in a light-free environment for 10 min; 200 µL solution B was added and incubated in a light-free environment for 10 min; and 200 µL solution C was added and incubated in at +4° C in a protected-light environment for 10 min. All analyses were performed by flow cytometry (BD Via, New Jersey, USA).

### 2.9. Acridine orange and ethidium bromide (AO/EB) staining assay

At 24 h after the plating of HeLa cells in 12-well plates at 5 × 10^4^ cell/mL, sulphonamide derivative compounds at concentrations of 10, 25 and 50 µM were added to the plates, and the cells were incubated at 37 °C for 24 h. The cells were then washed with PBS and released for incubation in a solution including 100 µL (acridine orange (100 µg/mL) and ethidium bromide (100 µg/mL) at room temperature for 5 min. The morphological fluctuations indicating apoptosis were investigated under florescent microscopy (Olympus CKX53, DP73, Japan).

### 2.10. Detection of DNA damage (γ-H2AX) by immunofluorescence staining

HeLa cells (1 × 10^4^/mL) were seeded in 12-well cell culture dishes and after 24 h, they were administered with the sulphonamide derivative compounds at doses of 10–25–50 µM and incubated for 24 h. The cells were washed with PBS and fixed with 100% methanol at −20 °C overnight, which was followed by PBS washing three times for 10 min for each washing. The cells were treated with 0.2% Triton X-100 in a shaker, at room temperature for 5 min. They were washed with PBS, and 1% BSA-containing PBS was then added, and incubation with primary mouse monoclonal anti-γ-H2AX antibody (Cell Signaling Technology, Danvers, MA) was applied at 37 °C for 1 h. The cells were washed with PBS for 5 min three times. Secondary goat anti-mouse Alexa-488-conjugated IgG (Invitrogen, Thermo Fisher Scientific, USA) was administered and the cells were incubated at 37 °C for 20 min, followed by PBS washing for 4–5 min three times. 1 mL 70% EtOH was added and, at +4 °C, released to incubation for 5 min. 1 mL 100% EtOH was added in a shaker, which was then incubated at room temperature for 1–2 min. The nucleus was stained with DAPI and the images were recorded using an Olympus Inverted Fluorescence Microscope (Olympus CKX53, DP73).

### 2.11. Western blot analysis

The cells were seeded on 6 cm^2^ cell culture dishes and treated with sulphonamide compounds, at doses of 10–25–50 µM, for 24 h. The cells were then washed with cold PBS, which was followed by lysis with RIPA lysis buffer (10 mM Tris-HCl pH = 8, 1 mM EDTA, 1 mM EGTA, 140 mM NaCl, 1% Triton X-100, 0.1 SDS, 0.1% sodium deoxycholate), 1X phosphates and protease inhibitor (Santa Cruz Biotechnology, Dallas, USA). The acquired supernatants from the lysates which were centrifuged at 12,000 *g*, + 4 °C, for 10 min, were transferred to new tubes. Protein concentration was detected using the protein assay kit BCA (Thermo Fisher Scientific, Waltham, MA). After a 30-min period of protein (50 µg) standing in 10% SDS–PAGE gel at 50 V and at 80 V for 3 h, those were blotted to PVDF membrane at 70 V for 5 min (Bio-Rad Turbo Transfer System). After embedding in 1X TBST (12.1 g Tris, pH 7.5, 70 g NaCl, 0.1% Tween-20) containing 5% dry milk or 5% BSA, the primer monoclonal antibodies shown in Table 2 were applied to the membrane and left overnight, which was followed by washing with 1 X TBS-T and an incubation period with secondary antibodies of HRP rabbit or mouse (1/10.000) for 60 min. The membrane was again washed with 1 X TBS-T. For band imaging, ECL substrate (EMD Millipore Corp., Billerica, MA) was added and observations were made via the imaging system (LI-COR Odyssey Fc).

**Table 2.** List of antibodies used for the Western blot.

Antibody	Brand	Dilution ratio	Rabbit/Mouse	Time
Cleaved caspase-3	ST John’s Laboratory	1/1000	Rabbit	Overnight +4 °C
Cleaved caspase-8	ST John’s Laboratory	1/1000	Rabbit	Overnight +4 °C
Cleaved caspase-9	ST John’s Laboratory	1/1000	Rabbit	Overnight +4 °C
Cleaved-PARP	Millipore	1/1000	Rabbit	Overnight +4 °C
CA-9	Cell Signaling	1/1000	Rabbit	Overnight +4 °C
Beta-actin	Cell Signaling	1/50000	Mouse	1–2 h +4 °C
Anti-rabbit IgG HRP	Cell Signaling	1/10000	Rabbit	1–2 h +4 °C
Anti-mouse IgG HRP	Santa Cruz	1/10000	Mouse	1–2 h +4 °C

### 2.12. Real-time quantitative PCR

All gene expression levels of the cells were examined following 24-h incubation with sulphonamide compound 2 at a dose of 25 µM. Total RNAs were isolated using the miRNeasy mini kit (Qiagen, Hilden, Germany) and reverse transcription was performed with the Ipsogen RT Set (Qiagen, Hilden, Germany) according to the kit protocol. RT-qPCR was then performed using the QuantiTect SYBR Green PCR kit (Qiagen, Hilden, Germany) in the Rotor-Gene Q real-time PCR system (Qiagen, Hilden, Germany). Each sample was studied in triplicate, using primer sets (Table 2) and GAPDH. Gene expressions were calculated with the 2^−ΔΔCt^ method and were compared to control groups. The GraphPad Prism 6 program defined the *p* values. Primers were designed using Primer blast on the National Center for Biotechnology Information website (https://blast.ncbi.nlm.nih.gov/Blast.cgi). All primers were determined to be 95–100% efficient and all exhibited only one dissociation peak. The sequences are listed in Table 3.

**Table 3.** List of primers used for real-time PCR.

Primer	Forward(5′-3′)	Reverse(3′-5′)
Caspase-3	GAGCACTGGAATGTCATCTCGCTCTG	TACAGGAAGTCAGCCTCCACCGGTATC
Caspase-8	CATCCAGTCACTTTGCCAGA	GCATCTGTTTCCCCATGTTT
Caspase-9	ATTCCTTTCCAGGCTCCATC	CACTCACCTTGTCCCTCCAG
Caspase-12	GCCATGGCTGATGAGAAACCA	TCGCATCCCCAAAAGGTCAA
CA IX	AGTCATTGGCGCTATGGAGG	TCTGAGCCTTCCTCAGCGAT
NRF-2	TTCGGCTACGTTTCAGTCAC	TCACTGTCAACTGGTTGGGG
BAX	TCCATTCAGGTTCTCTTGACC	GCCAAACATCCAAACACAGA
BCL-2	ATCGTCGCCTTCTTCGAGTT	ATCGTCGCCTTCTTCGAGTT
LC3	ATCATCGAGCGCTACAAGGG	AGAAGCCGAAGGTTTCCTGG
Beclin-1	CGACTGGAGCAGGAAGAAG	TCTGAGCATAACGCATCTGG
GAPDH	GGAAGGACTCATGACCACAGT	GGATGATGTTCTGGAGAGCCC

### 2.13. Measurement of intracellular free amino acids by LC-MS/MS

The intracellular free amino acid level was measured according to the Chen et al. method^4^. HeLa cells were planted in 10 cm^2^ cell culture dishes, and following 24-h treatment with sulphonamide at a dose of 25 µM, the growth medium was poured out, and the cells were swiftly washed twice with 5 mL cold PBS. Pre-cooled MeOH (−60 °C) was added, and the cells were stripped with a cell scraper. The cell suspension obtained was replaced in 15-mL conic tubes. The samples were kept in liquid nitrogen for 10 min and then dissolved on ice, which was triplicated until the cells were shredded. The samples were centrifuged at 3000 *g* at 4 °C, for 30 min, and the supernatants were transferred to new tubes. The amino acid level in the supernatant was measured using LC-MS/MS according to the protocol of the Jasem kit. The Jasem-free amino acid assay kit is used for studies involving the diagnosis of various hereditary metabolic disorders and the feeding of newborns with hereditary metabolic disorders. In this study, the protocol used to determine the intracellular free amino acid is as follows. In a new tube, 50 µL supernatant, 50 µL internal standard solutions and 700 µL reagent 1 were mixed by vortex for 10 s, and the acquired solution was centrifuged at 4000 rpm for 5 min. Twenty-seven amino acids in the acquired supernatant were analysed in HPLC vials using LC-MS/MS (Shimadzu 8045, Japan). The residual pellet was lysed in 1 mL lysis buffer, protein concentration of which was detected using the BCA protein assay kit (Thermo Fisher Scientific, Waltham, MA). Finally, the total protein levels were normalised and the net amino acid levels in the supernatants were defined.

## 3. Results

### 3.1. Growth inhibition and cell viability

The time and dose-dependent cytotoxic effects on cancer (HT-29, HeLa, MDA-MB-231) and normal cells (HEK-293 and PNT-1A) of synthesised seven sulphonamide derivatives determined with the feature of CA IX enzyme inhibitor in a study by Durgun et al.^16^^,^^17^ were examined with the WST-1 method. The values of the compounds and 5-Fu IC_50_ used as positive control are shown in Table 4.

**Table 4.** Cytotoxicity of derivatives of sulphonamide on tumour cell lines and normal cell lines.

Compound	IC_50_ (*µ*M)^a^														
	Cancer cell									Normal cell					
	HeLa			HT-29			MDA-MB-231			HEK-293			PNT-1A		
	24 h	48 h	72 h	24 h	48 h	72 h	24 h	48 h	72 h	24 h	48 h	72 h	24 h	48 h	72 h
**1**	19.9 ± 1.1	18.8 ± 1.1	11.3 ± 1.1	46.1 ± 5.4	43.8 ± 1.2	107.6 ± 34.5	598.4 ± 125.4	68.8 ± 4.65	56.7 ± 3.55	59.7 ± 11.2	83.6 ± 21.2	35.8 ± 11.2	221.2 ± 12.2	86.2 ± 12.1	197.4 ± 11.2
**2**	27.1 ± 1.3	49.6 ± 0.8	255.8 ± 32.2	9355.7 ± 25.4	4232.7 ± 12.2	926.3 ± 54.3	1232.9 ± 67.5	951.5 ± 76.5	370.9 ± 23.4	113.9 ± 3.44	305.8 ± 12.3	75.2 ± 5.6	339.5 ± 32.2	641.3 ± 10.2	470.5 ± 10.2
**3**	36.3 ± 0.2	29.8 ± 2.3	367.6 ± 23.2	2421.1 ± 32.2	1050.5 ± 23.2	1365.0 ± 45.4	1786.4 ± 65.4	80.6 ± 12.4	96.7 ± 33.4	196.5 ± 14.3	268.5 ± 14.3	37.9 ± 3.6	700.8 ± 74.33	753.3 ± 6.5	3001.9 ± 45.4
**4**	262.5 ± 13.2	326.4 ± 23.3	244.7 ± 24.3	33.0 ± 2.3	23.7 ± 2.2	118.9 ± 23.2	232.7 ± 13.5	690.5 ± 34.5	178.3 ± 11.2	639.4 ± 12.3	215.9 ± 23.2	96.2 ± 12.2	403.5 ± 84.3	1074.0 ± 24.3	294.9 ± 35.4
**5**	373.8 ± 11.1	182.1 ± 12.2	211.0 ± 12.2	2811.2 ± 45.3	19741.8 ± 34.3	6330.6 ± 124.4	512.9 ± 65.4	74.7 ± 12.4	6232.4 ± 342.2	261.6 ± 20.5	1445.3 ± 25.4	170.2 ± 11.2	626.2 ± 55.4	1336.9 ± 45.4	1328.3 ± 1345.4
**6**	235.3 ± 10.2	198.3 ± 14.3	123.4 ± 11.1	345.4 ± 9.3	324.3 ± 22.3	187.3 ± 23.4	450.3 ± 123.2	340.3 ± 45.4	300.3 ± 12.3	340.3 ± 28.6	230.3 ± 56.7	100.2 ± 22.2	154.3 ± 16.5	120.2 ± 11.2	76.43 ± 13.3
**7**	546.3 ± 23.2	347.5 ± 10.2	210.2 ± 10.2	453.2 ± 12.2	249.3 ± 16.4	200.2 ± 19.3	234.3 ± 23.4	123.2 ± 12.3	98.2 ± 11.2	120.2 ± 13.8	98.3 ± 11.2	85.4 ± 11.2	250.3 ± 13.55	190.3 ± 8.6	130.2 ± 32.2
**5-FU**	37.8 ± 4.3	31.2 ± 2.3	14.7 ± 0.6	47.3 ± 1.2	23.4 ± 2.1	19.3 ± 1.2	42.6 ± 2.56	37.9 ± 12.3	21.0 ± 4.3	44.6 ± 7.2	19.8 ± 2.3	14.5 ± 3.2	23.1 ± 4.55	21.2 ± 2.2	17.5 ± 7.6

^a^Values are means of three independent experiments.

As a result of the WST-1 analysis, while compound **1** of the seven sulphonamide derivative substances was observed to have the strongest cytotoxic effect in 72 h on HeLa cells with a high level of CA IX expression; it was seen to be less effective on other cells (Table 4). Compound **1** was also seen to be more effective on the HeLa cells than 5-Fu. Therefore, of all the substances, as compound **1** showed the strongest and selective cytotoxic effect on HeLa cells, the apoptotic effect mechanisms on cervical cancer cells with high CA IX expression (HeLa) and the relationship of this effect with CA IX were investigated.

### 3.2. The effects of compound 1 on HeLa cell proliferation

**Figure 1.**
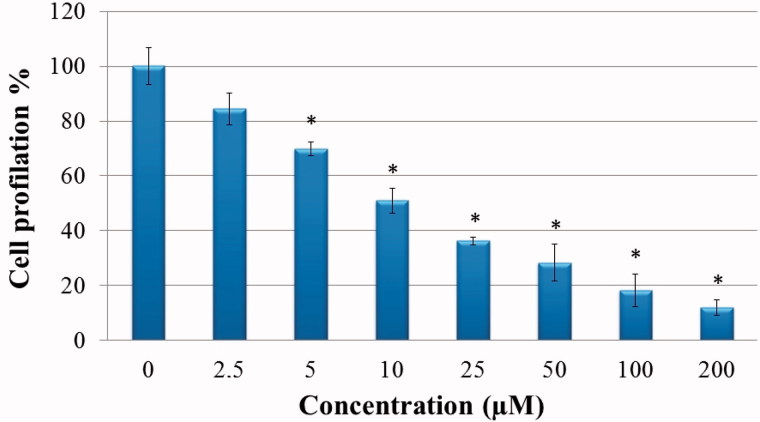
Antiproliferative effect of sulphonamide 1 against cervical cancer cell (HeLa) growth *in vitro*: HeLa cells grown in 96-well plates were treated with various concentrations of compound **1** (0–200 µM) diluted in the RPMI media for 24 h. All data are expressed as mean ± SD values from three independent experiments. *p < 0.01, compared with the control group, are considered significant.

The effects of compound **1** on HeLa cell proliferation were examined with the BrdU ELISA method. Compound **1** was determined to show a dose-dependent, strong, antiproliferative effect on HeLa cells (Figure 1).

### 3.3. The effects of compound 1 on HeLa cell apoptosis

**Figure 2.**
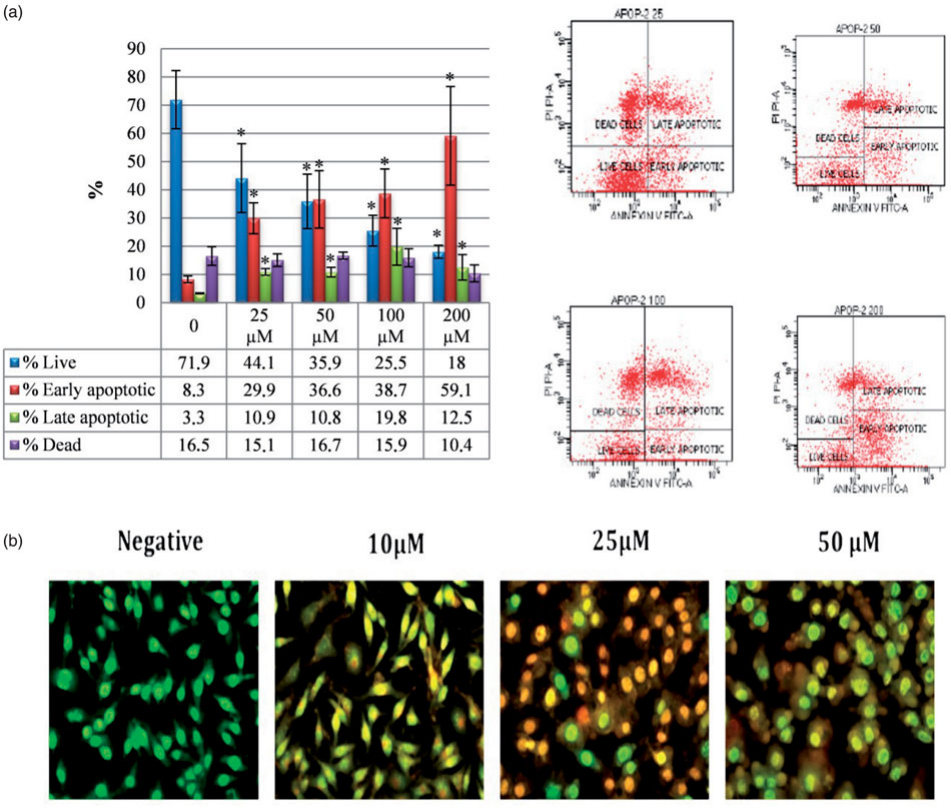
Induction of apoptosis in HeLa cells by sulphonamide 1. (a) The apoptosis ratio analysed by flow cytometry data analyses of HeLa cells and contour diagram of Annexin-V/PI flow cytometry, (b) morphological changes observed under florescence microscope in compound **1** treated HeLa cells after staining with AO/EB. All data are expressed as mean ± SD values from three independent experiments. *p < 0.05, compared with the control group, are considered significant.

Sulphonamide compounds are known to be cytotoxic to cancer cells by inducing apoptosis^18^. In this study, to confirm that compound **1**, which showed the strongest cytotoxic effect, induced apoptosis, the Annexin-V FITC test was performed as an indicator of early apoptosis. The HeLa cell line was treated with compound **1** at doses of 10–25–50 µM. The flow cytometric Annexin-V FITC models of the HeLa cancer cells are shown in Figure 2(a). It was determined that compound **1** showed an apoptotic effect from 25 µM onwards and as the dose increased, so the apoptotic effect increased. Most of the apoptotic effect was at the stage of early apoptosis (29.9%–59.1%) and the cell death rate (10%–16.7%) was determined to be at the same level as in the control group (Figure 2(a)). Compound **1** also significantly increased the cleaved caspase-3 level in HeLa cells and these findings were confirmed morphologically in the AO/EB results (Figure 2(b)).

### 3.4. Acridine orange/ethidium bromide (AO/EB) double staining

The AO/EB double staining method was applied to observe the morphological changes in cell death. AO/EB staining revealed the morphological changes of apoptosis in the HeLa cells treated with compound **1**. As seen in Figure 2(b), the nonapoptotic control cells were stained green and in the apoptotic cells, yellow/red colours emerged due to the fragmentation of the nuclear DNA in the nuclei.

### 3.5. The effects of compound 1 on pHi, pHe and lactate level

**Figure 3.**
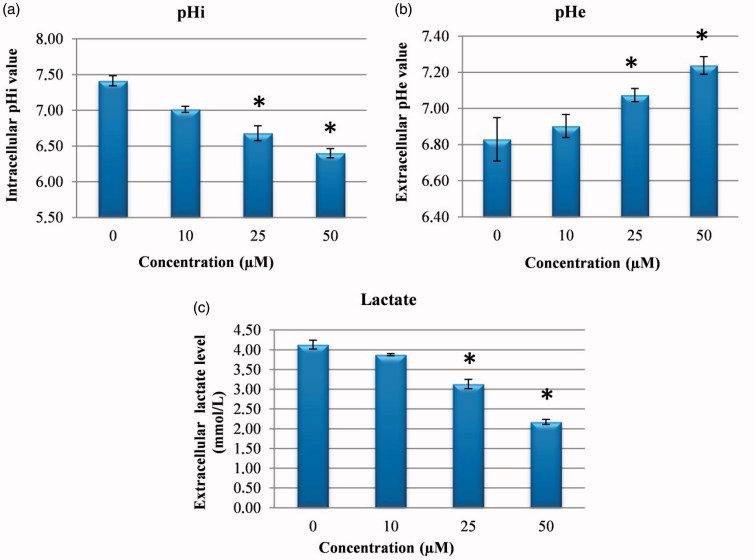
Measurement of pHi, pHe and extracellular lactate level. (a) The effects of sulphonamide **1** on pHi in HeLa cell. (b) The effects of compound **1** on pHe in HeLa cell media. (c) The effects of sulphonamide **1** on lactate level in HeLa cell media. The data are presented as the mean ± SD (*n* = 3). *p < 0.01 compared with the control.

CA IX has been shown to have a significant role in protecting the cytosol against intracellular acidosis, thereby allowing survival and proliferation of the cell even in hypoxic conditions^7^. Therefore, it was examined whether CA IX inhibition in cancer cells could intervene in pHi, pHe and lactate level. It was shown that treatment of HeLa cells with compound **1** CA IX inhibitors caused a significant dose-related fall in pHi and lactate level of media and a significant increase in pHe (Figure 3). It has been shown that pHi has an important role in homeostatic balance in cancer cells, just as in normal cells, and changes in the pH of HeLa cells lead to an increase in ROS and DNA damage by impairing MMP and thus induce cellular apoptosis^19–21^. Similarly, as pHe plays an important role in the metastatic progression of many cancers, CA IX continuously contributes to the maintenance of the slightly alkaline pHi in cancer cells, whereas it decreases the external pHe. The results obtained in this study confirmed that the reducing extracellular lactate and pHi levels and increased pHe are a consequence of CA IX inhibition by compound **1**^22^.

### 3.6. Compound 1 induces apoptosis through the extrinsic and intrinsic mitochondrial pathway

Apoptosis may start through intrinsic or extrinsic pathways^23^^,^^24^. To better identify the initial pathway of apoptosis, the Western blot for apoptosis regulators was applied. The presence of cleaved caspase-3, caspase-8, caspase-9, and cleaved-PARP was investigated to determine by which route cellular apoptosis had occurred. The CA IX level was also examined to partially determine the CA IX inhibitor property of compound **1**.

**Figure 4.**
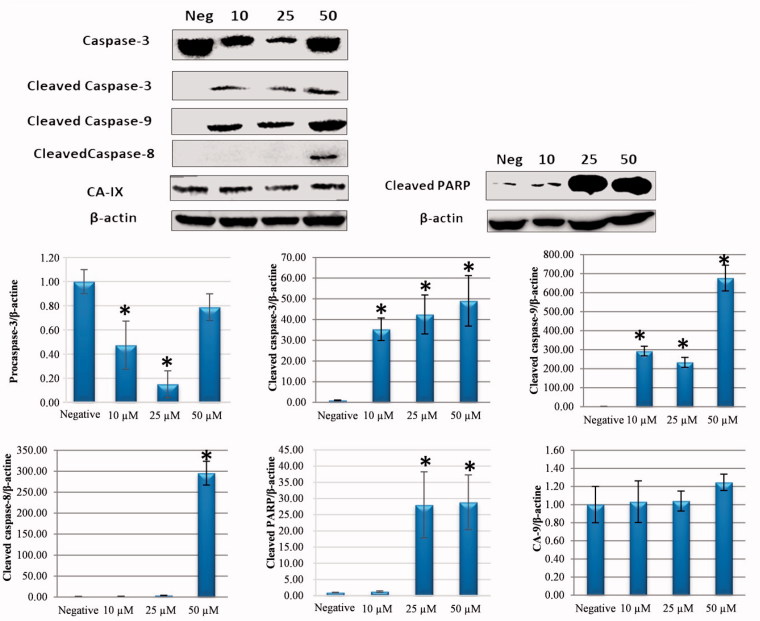
Effect of sulphonamide 1 on apoptosis signalling proteins (cleaved caspase-3, caspase-8, caspase-9, and PARP) and CA IX expression levels in HeLa cells. Cells were treated with compound **1** (0, 10, 25 and 50 µM) for 24 h. Proteins were normalised to the respective β-actin and are presented relative to the value for the untreated control cells. The densitometry quantification of blot was determined by software (Li-Cor Fc). The data are presented as the mean ± SD (*n* = 3). *p < 0.05 compared with the control.

When the cleaved caspase-9 and caspase-8 levels were examined, which provide determination of the main pathways of apoptosis, it was determined that the internal apoptosis pathway was triggered through an increase in the cleaved caspase-9 level associated with an increased dose of compound **1** and the external apoptosis pathway was triggered associated with an increase in cleaved caspase-8 at a dose of 50 µM (Figure 4). According to these results, it was determined that compound **1** caused cell death by activating both the intrinsic and extrinsic pathways.

The mitochondrial apoptotic pathway is induced with cytochrome-c expression from the mitochondrial external membrane regulators, which are regulated by the Bcl-2 family. While anti-apoptotic Bcl-2 gene expression was reduced in HeLa cells treated with compound **1**, the apoptotic Bax gene expression increased (Figure 8). It was found that the compound **1** induces apoptosis through the mitochondria-dependent intrinsic pathway according to the results of increasing cleaved caspase-9 and caspase-3.

### 3.7. The effect of compound **1** on changes in mitochondrial membrane potential (MMP)

**Figure 5.**
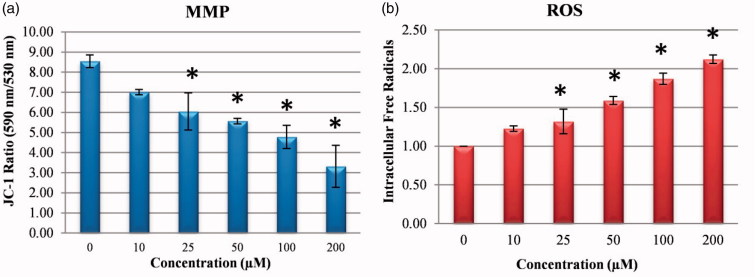
Reactive oxygen species (ROS) levels and mitochondrial membrane potential (MMP) stability detected by spectroflourimetry. Cells were treated with various concentrations of compounds **1** (0–200 μM) for 24 h. (a) The cells were stained with the JC-1 dye and spectroflourimetry was performed to determine the MMP stability. (b) The cells were stained with the ROS red dye and spectroflourimetry was performed to determine the intracellular ROS. The data are presented as the mean ± SD (*n* = 3). *p < 0.05 compared with the control.

MMP loss has been reported to be related to the start and activation of some apoptotic cascades^25^^,^^26^. To examine changes in MMP after treatment with compound **1**, MMP was measured with the JC-1 method. As seen in Figure 5(a), MMP was determined to have reduced in a dose-dependent manner after treatment with compound **1**.

### 3.8. The effect of compound 1 on changes in intracellular free radicals (ROS)

A reduction in pHi and a significant increase in cellular ROS production have been revealed to mobilise a “signal molecule” for the activation of MAPK associated with the pH of ROS^27^^,^^28^. In the current study, when the effect of compound **1** was examined on the intracellular ROS amount which occurred associated with the decrease in pHi in the HeLa cells, there was a dose-related increase in the amount of ROS (Figure 5(b)). In addition, the increase occurring in the NRF-2 gene level (Figure 8), which triggers the intracellular antioxidant system, confirmed the increase in intracellular ROS.

### 3.9. The effect of sulphonamide compound 1 on DNA damage (γ-H2AX)

**Figure 6.**
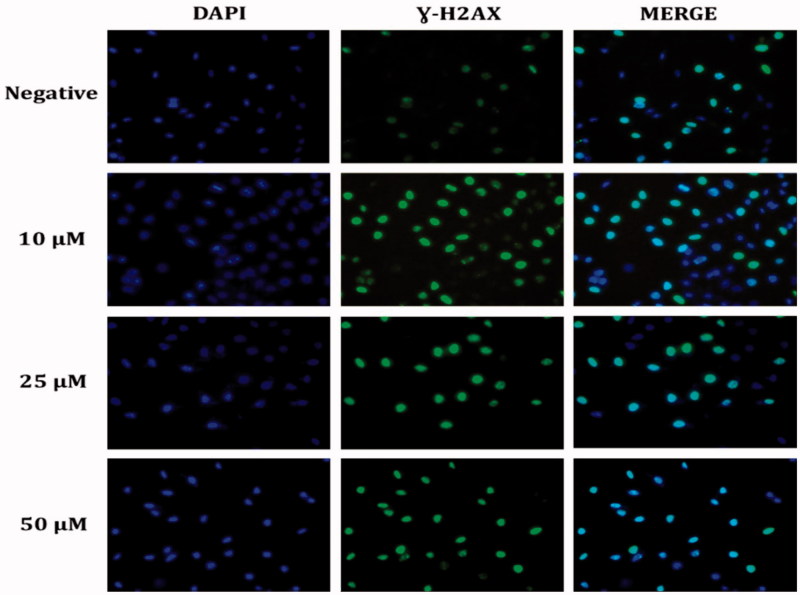
Representative DNA damage microscopy images from HeLa cell treated with sulphonamide **1**. A fragment of each cell was fixed and processed for γ-H2AX immunofluorescent staining (IFA). γ-H2AX staining is green; nuclei are stained with DAPI blue.

DNA damage developed with the increase in ROS in HeLa cells. To investigate DNA damage, immunofluorescent imaging analysis of γ-H2AX focus points in the nucleus was used to reveal the extent of the damage leading to apoptosis. The results showed that the treatment with compound **1** increased the absence of γ-H2AX in the nucleus and the effects throughout 24 h following treatment were dependent on the concentration in the HeLa cells (Figure 6). The increasing γ-H2AX focus points in the DNA with compound **1** treatment provided further evidence that the damage in the DNA of the HeLa cells originated from ROS production (Figure 5(b)).

### 3.10. Cell cycle-perturbed distribution of HeLa cells treated with compound 1

**Figure 7.**
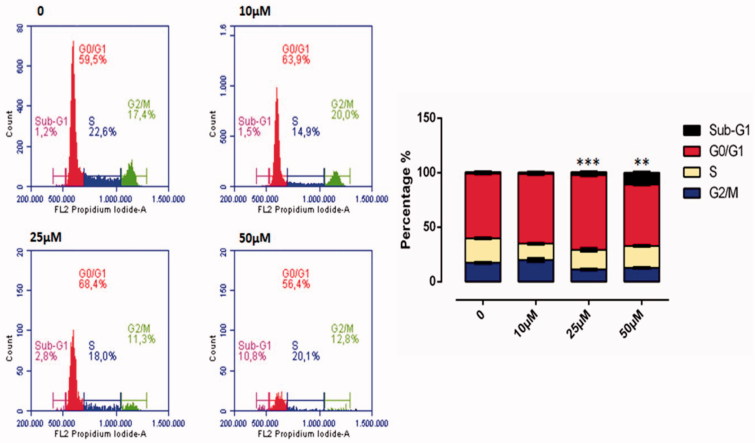
Cell cycle analysis of HeLa cell cancer treated with sulphonamide **1** at 0, 10, 25 and 50 µM concentration treatment after 24 h. All data are expressed as mean ± SD values from three independent experiments. *p* values of less than 0.05 (**p < 0.05, ***p < 0.01, compared with the control group) are considered significant.

ROS accumulation plays an important role in the initiation and maintenance of the apoptosis and cell cycles in cancer cells. The flow cytometric cell cycle patterns of the HeLa cells treated with compound **1** are shown in Figure 7. Sub-G1 populations were higher in HeLa cells treated with compound **1** than in the control cells (Figure 7). The cell division effect of compound **1** on the HeLa cells was seen from 25 µM onwards and cell division was determined to have slowed related to an increase in dose. At 50 µM dose, cell cycle stages were determined as 10.8% in the Sub-G1, 56.4% in the GO/GI phase, 20.1% in the S phase and 12.8% in the G2/M phase. When the 50 µM dose was compared with the control group, it was observed that after application of the substance, cells in Sub-G1 phase were halted (Figure 7).

### 3.11. The measurement of gene expression levels

**Figure 8.**
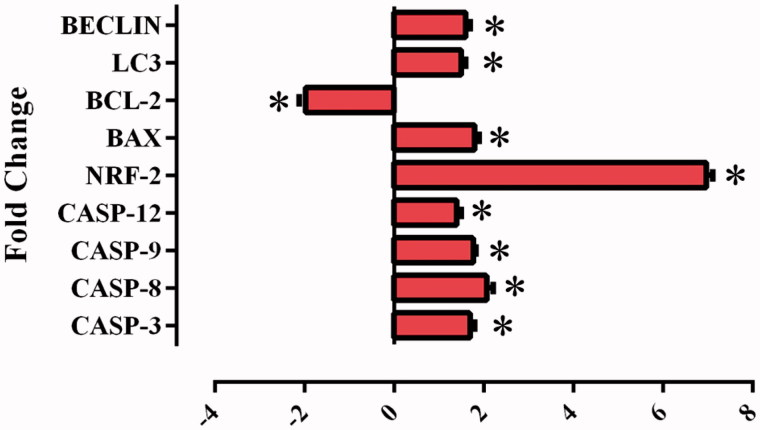
Effects of sulphonamide 1 (25 µM)) on the expression of apoptotic (caspase-3, caspase-8, caspase-9, caspase-12 and Bax), anti-apoptotic (Bcl-2) and autophagy (LC3, Beclin) related genes. The data are presented as the mean ± SD (*n* = 3). *p < 0.01 compared with the control.

To confirm the results found in the PCR method based on partial amounts, the real-time PCR (qRT-PCR) method was used in this study. Using mRNA samples isolated in the HeLa cells incubated for 24 h with a 25 µM dose of the sulphonamide derivative compound **1** used in the cell vitality and proliferation experiments, the gene expression levels were examined of the genes (caspase-3, caspase-8, caspase-9, and caspase-12, Bcl-2, Bax, Lc3, Beclin-1) associated with the apoptosis and autophagy pathways and NFR-2 gene (Figure 8).

Increases observed in the levels of the genes which have a role in the apoptosis pathway (caspase-3, caspase-8, caspase-9, and caspase-12 and Bax) in the HeLa cells after application of compound **1** (*p* < 0.01) showed triggering of the apoptosis pathway. The synthesised substances thought to be potential CA IX inhibitors showed a statistically significant increase in the intracellular ROS in the level of NFR-2 gene (*p* < 0.01). These data showed a correlation with ROS analysis.

### 3.12. The effect of compound 1 on the intracellular free amino acid level

**Figure 9.**
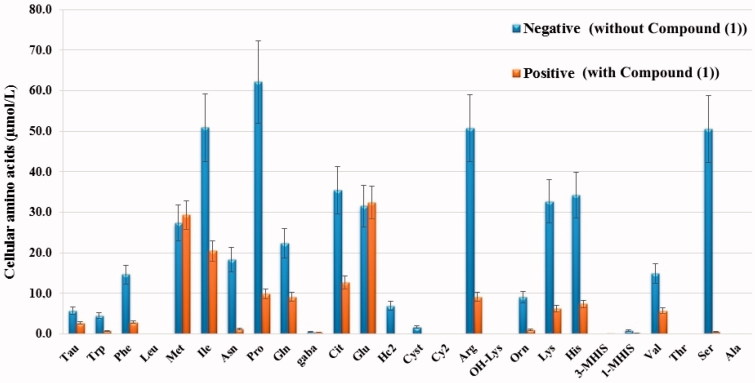
Intracellular free amino acid profiling of HeLa cells: The absolute levels of intracellular free amino acids were quantified using LC-MS/MS. All data are presented as mean ± SD values (*n* = 3). Error bars indicate the SD.

The change occurring in the intracellular 27 free amino acid levels in the HeLa cells after application of the substance was examined with LC-MS/MS (Figure 9). HeLa cells treated with compound **1** were identified as positive and those not applied as negative group. When we compare amino acid levels in HeLa cells, the levels of intracellular free amino acids in the positive group were decreased relative to those of the negative group.

## 4. Discussion

Some new inhibitors such as sulphonamides with high affinity for CA IX have been determined to induce apoptosis by reducing pHi and proliferation in cancer therapy^8^^,^^18^^,^^29^^,^^30^. Therefore, various studies have been conducted for the synthesis of analogues and derivatives of sulphonamides because of this CA IX inhibition property to further increase the anticancer potential and reduce the toxic effects on normal cells^1^^,^^8^^,^^31–35^.

In a previous study by the current authors, various derivatives of sulphonamides were synthesised to produce a greater CA IX inhibitor property and stronger bioactive compounds, and in comparison with traditional CA inhibitors such as acetazolamide (AAZ), these substances were shown to have higher selectivity against CA IX^16^^,^^17^. In the current study, the effects of these substances were examined for the first time on various cancer cell lines in respect of their ability to inhibit cell proliferation and for selective death in HeLa cells with CA IX expression. The primary aim of the study was to examine dose and time-related cytotoxic effects of seven sulphonamide compounds on human cancer cells (HeLa, HT-29 ve MDA-MB-231) and normal cell lines (HEK-293, PNT-1A).

Among the seven sulphonamide derivatives used in this study, compound **1** showed the strongest cytotoxic effect on HeLa cells with high CA IX expression. While compound **1** applied to HeLa cells inhibited proliferation in a dose-related manner with an IC_50_ value (11.3 µM) lower than that of 5-Fu (14.7 µM) which is used in chemotherapy, much lower cytotoxicity was seen in normal cells. Furthermore, when growth was not affected in normal cells (HEK-293, PNT-1A) and in cancer cells such as MDA-MB-231, compound **1** selectively inhibited HeLa cell growth. These results suggest that CA IX inhibitors could have a role in the mediation of the antitumour effect of CA inhibition. Similar to the results obtained in the current study, previous studies have shown that various CA inhibitors induced apoptosis and inhibited the invasive capacity of cancer cell lines with high CA-II, CA IX and CA-XII levels^18^^,^^36^. Small structural changes in the R moiety of the synthesised compounds **1–7** lead to the changes in the cytotoxicity properties. Since the variation of cytotoxicity values for compounds^2–7^ is minimal (HeLa 72 h), it is difficult to perform SAR evaluation. The most remarkable situation of the compound **1** is that the cytotoxicity effect in the presence of a bromine atom is 10–34 times higher than the other compounds **2–7**.

Based on the data of growth inhibition, compound **1** was selected to test the apoptotic effects on CA IX-positive cervical cancer cells (HeLa). To understand the dynamics of the molecular changes related to apoptosis and the phenotypic changes observed in the cancer cells, cleaved caspase-3, caspase-8, caspase-9, and cleaved-PARP, CA IX protein level, γ-H2AX, pHi, pHe, lactate level change, MMP, ROS activity, cell cycle distribution, amino acid metabolism and gene expression analyses were used. Annexin-V, AO/EB and cleaved caspase-3 activation (a sign of apoptosis) were observed in the HeLa cells. The results of the study showed that apoptosis was started through mediation of caspases in the HeLa cells by compound **1**.

Cellular acidosis is a trigger of the early stage of apoptosis and it has therefore been assumed that pHi is kept high to prevent apoptosis in cancer cells^27^^,^^29^^,^^37^. By inducing activation of endonuclease II, a reduction in pHi induces cellular apoptosis by DNA fragmentation and MMP impairment, leading to an increase in ROS^38–43^.

In a study by Matsuyama et al.^44^, it was reported that cytochromic acidification to apoptotic stimuli associated with the mitochondria such as staurosporine and ultraviolet light and cytochrome-c expression stimulated caspase activation and mitochondrial depolarisation and therefore, with the impairment of intracellular homeostasis and metabolism by drugs which reduce the pHi in cancer cells, apoptosis is expected to form in cancer cells^44^. In a similar study, the decrease in pHi occurring as a result of CA IX inhibition was determined to cause apoptosis of tumour cells by causing an increase in ceramide amount, upregulation of p38-MAPK activity and stimulation of oxidative stress^18^. In the current study, despite a reduction in pHi with sulphonamide **1** treatment, there was an increase in pHe and ROS leading to MMP loss. These results were similar to those of previous studies and are an important indicator that sulphonamide **1** showed an apoptotic effect on CA IX inhibition.

An increasing amount of ROS lead to DNA damage which leads to an increase in p53 expression. The activation of p53 activates the intrinsic apoptotic pathway by triggering the permeability of the outer mitochondrial membrane and coordinating pro-apoptotic Bax and anti-apoptotic Bcl-2. The activated intrinsic pathway is very important in starting apoptotic cell death under the effect of ROS. By activating the apoptotic protease activating factor 1/caspase-9 apoptosome complex, cytochrome-c that is expressed causes activation of cleaved caspase-3 that is critical for apoptosis^45–48^.

The results of this study showed that by impairing MMP, the application of compound **1** on human HeLa cells caused an increase in ROS, thereby creating oxidative stress and causing apoptotic cell death. In addition, after 24 h of treatment with sulphonamide **1**, intrinsic apoptosis was shown to have been triggered by activating the fragmentation of procaspase-3 and PARP fragmentation, thereby increasing the levels of cleaved caspase-3 and cleaved caspase-9 protein expression (Figure 4). Moreover, compound **1** was found to induce the intrinsic apoptosis pathway by increasing cleaved caspase-9 level at low doses (10–25 µM). The increase in cleaved caspase-8 and CA IX protein expression that occurred at a dose of 50 µM was observed to have activated the extrinsic apoptotic pathway associated with CA IX (Figure 4).

In the light of the interaction of the molecular markers of apoptosis explained above, it was observed in the current study that Bcl-2 was downregulated, and proapoptotic caspase-3, caspase-8, caspase-9, caspase-12 and Bax were upregulated. The Bcl-2 protein family controls the sensitivity of apoptosis as a regulator of MMP. After impairment of MMP, the mitochondrial permeability pores will open to allow the flow of apoptotic factors^49^. By upregulating following treatment, the Bax/Bcl-2 ratio, Bax gene expression increases and Bcl-2 gene expression decreases and as a result, caspase-3 mobilises to trigger a signalling cascade as an effector of the apoptosis pathway (Figure 8). In addition, a significant increase was determined in NRF-2 gene expression (Figure 8). NRF-2 is an important transcription factor that regulates a broad gene sequence for antioxidant and detoxification enzymes and protects cells by activating the antioxidant system against an increase in free radicals^50^. The increasing NRF-2 level determined in the current study was confirmed by the increasing intracellular ROS level.

ROS accumulation plays an important role in the onset of the apoptosis and cell cycle stoppage in cancer cells^51^. An increasing amount of ROS leading to DNA damage leads to an increase in p53 expression^52^. The activation of p53 following DNA damage causes the cell cycle to halt and the emergence of several proteins that are important for repair and the control of apoptosis^53^. Holding the cell at the G1 control point in the cell cycle is very beneficial in the prevention of continuing proliferation of cancer cells^54^. These data showed that sulphonamide **1** triggered the halting of the G1 phase in HeLa cells. G1 holding is a well-known result of DNA damage related to p53 induction. It was confirmed in the immunofluorescence analysis that there was DNA damage in the increasing γ-H2AX foci (Figure 6) and an increase in the cleaved-PARP level in the HeLa cells treated with compound **1** (Figure 4).

In addition, the increasing p53 associated with the increase in ROS is known to mediate apoptosis by holding the cell cycle in the G1 stage and by inhibiting the PI3K/AKT/mTOR pathway^54^. The production of amino acids is prevented in mTOR inhibition. Amino acid deficiency provides a suppression signal that covers more than others such as the insulin signal of the mTORC1 pathway. mTOR supports anabolic metabolism and inhibits autophagy induction. The intracellular amino acid level is a signal necessary for the regulation of mTOR kinase activity. At the same time, mTOR kinase activity is controlled by growth factors^55^^,^^56^. As amino acids are both the building blocks of protein synthesis and mediating metabolites triggering other biosynthetic reactions, they have an important role in cellular metabolism^57^. While amino acids suppress autophagy, a lack of amino acids stimulates autophagy^58^. Amino acid deficiency provides a suppression signal inhibiting the mTORC1 pathway. Therefore, through the mTORC1 pathway, amino acids play a key regulatory role in cell proliferation^59^.

With autophagy management, cells can be brought to a reversible state necessary for biosynthesis with amino acids and other macromolecular materials^60^. In the autophagy process, when the amino acid concentration falls, autophagy provides amino acid production which is necessary for the survival of the cell. When the demand for amino acids is increased, autophagy is suppressed^61^. By inducing autophagy, the associated Beclin-1 and LC-3 genes can suppress tumour growth. Therefore, they are accepted as a potential therapeutic target in cancer management^62^^,^^63^. In the current study, there was a decrease in the amount of all the amino acids in the HeLa cells treated with sulphonamide **1** (Figure 9) and associated with that, it was seen that autophagy had increased Beclin-1 and LC3 gene expression (Figure 8).

In conclusion, the results of this study showed that as a result of CA IX inhibition by the sulphonamide derivative **1**, the change created in pH (pHi and pHe) was able to reduce cell proliferation and could induce apoptosis in cancer cells. One of the most important mechanisms in this potential antitumour effect is the ability of compound **1** to induce intracellular acidosis by suppressing CA IX activity. These findings indicate that CA IX inhibition could be a useful method in the clinical treatment of human tumours that express this enzyme at a high rate.
